# Sustainable health care in a renal centre - carbon saving is coupled with cost-efficiency

**DOI:** 10.1007/s40620-025-02354-x

**Published:** 2025-07-23

**Authors:** Stephanie Mei Yann Choo, Gareth Murcutt, Ingeborg Steinbach, John Stoves

**Affiliations:** 1 Department of Renal Medicine, St James’s University Hospital, Beckett St, Harehills, Leeds, LS9 7TF UK; 2 UCL Department of Renal Medicine, Royal Free Hospital London, London, NW3 2QG UK; 3 Centre for Sustainable Healthcare, 8 King Edward Street, Oxford, OX1 4HL UK; 4 Department of Renal Medicine, Bradford Teaching Hospitals NHS Trust, Duckworth Ln, Bradford, BD9 6RJ UK

**Keywords:** Sustainability, Green nephrology, Greenhouse gas emissions, Haemodialysis

## Abstract

**Background:**

Healthcare contributes significantly to global carbon dioxide equivalent emissions, with kidney care contributing disproportionately to this. Renal medicine was one of the first specialities to actively develop a "green" community. This paper is a retrospective review of a series of comprehensive and impactful green initiatives across various aspects of kidney care delivery in a kidney unit from 2007 to 2024.

**Methods:**

The interventions include using e-consultations and virtual clinics, online priming of haemodialysis machines, upgrade of water treatment systems, centralised dialysate acid delivery, use of 1:44 acid concentrate, use of dialysate autoflow function, installation of energy-efficient lighting, and incremental and decremental dialysis practices. Financial and environmental saving estimates for the haemodialysis-related interventions were calculated based on a 40-bed haemodialysis unit. A hybrid carbon footprinting approach was utilised to calculate the greenhouse gas and financial savings.

**Results:**

The cumulative estimated greenhouse gas and financial savings exceed 1,000 tonnes of carbon dioxide equivalent and £2.8 million, respectively. Among sustainable initiatives in haemodialysis, online priming, use of central acid delivery, dialysate autoflow facility, and incremental and decremental haemodialysis showed the most significant savings.

**Conclusions:**

Interventions to facilitate environmental sustainability may require upfront funding and staff investment of time and effort, but the dividend is long-term environmental protection, financial savings, enhanced quality of care, greater staff satisfaction and enhanced service resilience. Sharing these experiences may help other institutions to integrate green initiatives into everyday service planning.

**Graphical abstract:**

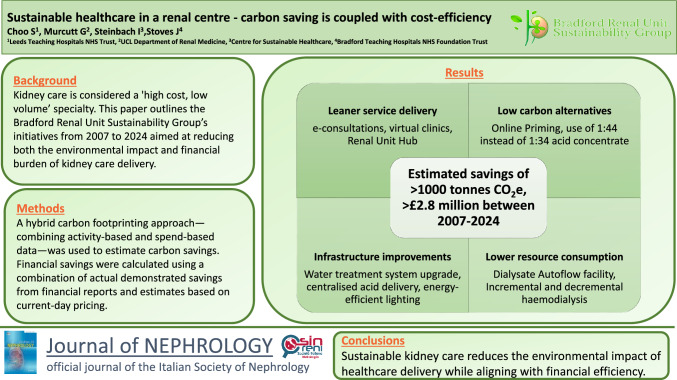

**Supplementary Information:**

The online version contains supplementary material available at 10.1007/s40620-025-02354-x.

## Introduction

Healthcare contributes to 4–5.2% of global carbon dioxide equivalent emissions [1, 2]. In 2019, the carbon footprint for the National Health Service (NHS) in England was estimated at 25 megatonnes of carbon dioxide equivalent [3]. Kidney care itself contributes significantly and disproportionately to healthcare emissions [4]. This is owing to higher carbon emissions as kidney disease progresses, driven by increased number of hospital attendances, medical treatment and the high carbon footprint of dialysis treatments [5]. Haemodialysis treatment requires significant water and energy consumption, consumables with single-use plastic, pharmaceuticals, medical equipment, and significant patient and staff travel, resulting in high greenhouse gas emissions ranging from 21.7 to 65 kg carbon dioxide equivalent per treatment [6–8].

In the United Kingdom (UK), the Green Nephrology programme was founded in 2009 to promote sustainable kidney care through the collaboration of various national renal societies and specialist NHS groups. The involvement of various stakeholders from the outset, including clinicians, healthcare managers, and engineers, working together to reduce the carbon footprint of renal services and promote eco-friendly innovations in the treatment of kidney disease, helped this movement gain rapid momentum in the UK [9]. The Bradford Teaching Hospitals NHS Trust’s nephrology department has been a firm supporter of the green nephrology movement and has been implementing sustainable initiatives since 2007. The aim of this study is to summarise a series of impactful green initiatives across various aspects of kidney care delivery in a single kidney centre from 2007 to 2024. During this period, the haemodialysis unit had an average capacity of 40 beds operating three shifts a day, six days a week.

## Methods

This paper is a retrospective review of sustainable interventions implemented by the Bradford nephrology department since 2007. These include using e-consultations and virtual clinics, online priming of haemodialysis machines, upgrade of water treatment systems, centralised dialysate acid delivery, use of 1:44 acid concentrate, installation of energy-efficient lighting, and incremental and decremental dialysis practices. Several key factors influenced the timing and selection of these initiatives, including the publication of the National Service Framework for Renal Services in 2005, which supported the introduction of e-consultation to facilitate care pathways for managing chronic kidney disease (CKD) in primary care. Advancements in dialysis machine functionality, coupled with a capital replacement programme and successful business cases facilitated service development. Initial successful sustainability initiatives led to the availability of project award funds for reinvestment into sustainability efforts. In addition, embarking on this sustainability journey resulted in regular exchange of best practices with other early adopters of sustainable healthcare approaches, particularly through collaboration with NHS Kidney Care and the Green Nephrology programme. Data on each intervention were gathered through a review of published case studies on the Centre for Sustainable Healthcare Green Nephrology resource library, local Trust records, and information provided by departmental staff.

The data were analysed within four main categories:

- Environmental savings: Reductions in water, energy, dialysate acid, and consumables with the corresponding greenhouse gas emissions.
- Financial savings: Actual or estimated financial savings of each intervention.
- Operational efficiency: Impact on staff and service delivery.
- Patient outcomes: Impact on patient care.

A hybrid carbon footprinting methodology to calculate the greenhouse gas savings was used, combining elements of both bottom-up and top-down approaches. It was chosen to balance the need for detailed, component-specific accuracy with the ability to derive meaningful estimates from aggregated, historical data. We used a range of data sources to calculate the greenhouse gas savings including the Department for Energy Security & Net Zero (DESNZ) and Department for Environment, Food & Rural Affairs (DEFRA) 2024 greenhouse gas conversion factors, published carbon footprint estimates of medical equipment, and expert opinion [10]. Calculations of the greenhouse gas savings are provided in the supplementary material.

The long-term, cumulative impacts of all interventions were calculated to estimate the total greenhouse gas and financial savings achieved between 2007 and 2024.

## Results

### Use of electronic referral systems, electronic consultations and virtual clinics

An electronic consultation (e-consultation) service was developed within SystmOne®, a clinical computer system used by many primary care practices in Bradford and Airedale [11]. Starting with 17 practices in 2007, it later expanded to all practices utilising SystmOne®. It allows general practitioners (GPs) to send e-consultations and share electronic health records (EHRs) with renal specialists. The specialist can access detailed clinical data, facilitating informed decision-making whether the patient requires clinic review, further tests or primary care management. Responses are recorded in EHRs and shared with referring teams, streamlining care and communication.

A Renal Unit Hub was subsequently created within the primary care EHR for advanced kidney disease patients to enhance the two-way sharing of information [12]. Since 2020, virtual clinics using secure phone and video services by NHS England’s Attend Anywhere service were introduced. This started with the virtual metabolic stone and young adult transition clinics, with plans to establish a virtual conservative care clinic in the future.

#### Outcomes

*Environmental Benefits:* When e-consultations were first introduced as a pilot, the unit received 68 e-consultations in a year [11]. As the pathway and service developed, this increased to 1200 per year in 2024, which now represents 60% of referral activity. Approximately 7,500 unnecessary face-to-face appointments have been avoided over an 18-year period, saving 165 tonnes of carbon dioxide equivalent.

*Cost Savings:* Between 2007–2021, a face-to-face appointment cost £135 per appointment based on estimates by the Personal Social Services Research Unit (PPSRU). By 2023, PPSRU reported that the cost of an average outpatient attendance had increased to £217 [13]. As there are no published financial analyses on the costs of e-consultations in the UK, we are unable to calculate the cost savings on these implementations accurately.

*Operational Improvement:* The e-consultation service facilitated prompt and informed decision-making, reducing the waiting time for specialist nephrology consultations. It also increased GP confidence in managing CKD. During the study period, the e-consultation service reduced face-to-face clinic referral rates by 78% compared to patients referred by letter, as nephrologists were able to better assess the appropriateness of a clinic review based on the clinical data. Avoiding unnecessary clinic appointments ensures that specialist unit resources are utilised for patients who need them the most [11]. The Renal Unit Hub reduced test duplication, improved shared care pathways such as hepatitis B vaccination rates and reduced administration time for the renal multidisciplinary team [12].

*Patient care:* Virtual appointments reduce disruptions to patients’ daily lives, minimising time off work or school and reducing transportation costs. GPs found that e-consultations increased their confidence and knowledge in managing CKD in the community. There was also more effective communication regarding patient care through e-consultations and the Renal Unit Hub [11, 12].

### Online priming

The unit implemented the use of online priming on haemodialysis machines in 2010. This involves the machine priming the bloodlines automatically with ultrafiltered dialysate, thereby avoiding the use of individual 0.9% saline infusion bags for priming, reinfusion or bolus [14, 15].

#### Outcomes

*Environmental Benefits:* Online priming reduces plastic waste from removing the use of saline infusion bags and intravenous giving sets, which reduces carbon emissions by 8.04 tonnes carbon dioxide equivalent/year.

*Cost Savings:* Removal of the saline infusion bags and giving sets would save £92,851 annually.

*Operational Improvements:* The automatic, online priming regimen reduces the workload for dialysis staff.

*Patient care*: Online priming reduces the risk of external contamination during the handling of the saline bag and giving set [15].

### Upgrade of water treatment systems

The initial two single-pass reverse osmosis water treatment systems were installed in 1995. In response to increased capacity requirements, two additional single-pass reverse osmosis units were installed in 2005 and 2007. The renal technologists found that the two older systems rejected 70% more water than the two newer ones. In 2011, £60,000 was spent to replace the older systems [16]. All four reverse osmosis systems wre thus of the same type and specification, and were configured to run in parallel, which ensured system redundancy.

#### Outcomes

*Environmental Benefits:* The updated systems save 8 million litres of water annually, reducing carbon emissions by 8.42 tonnes carbon dioxide equivalent in 2012 [16]. Using the updated 2024 DESNZ conversion factors, this would generate a saving of 2.7 tonnes carbon dioxide equivalent per annum.

*Cost Savings:* Reduced wasted water resulted in an annual saving of £18,000 in 2012 compared to the previous year [16]. Based on this estimate, the capital outlay for the project would be recouped in approximately 3.3 years. Under the trust cost improvement programme, the nephrology department budget benefited from 20% of these savings. Using 2024 pricing for water supply and sewerage, this would generate a saving of £24,240, annually.

*Operational Improvements:* The increased water flow to the haemodialysis unit supported future expansion and enhanced service resilience. Automated heat disinfections with the newer systems improved water quality and saved staff time.

### Centralised delivery of dialysate acid concentrate

In 2011, the haemodialysis unit installed a centralised dialysis acid delivery system to reduce acid concentrate and plastic waste. Acid was previously supplied in 6-L plastic canisters, resulting in an annual wastage of 50,142 L of acid and the disposal of 29,540 canisters [17].

The new two-loop system, with installation costs of £43,900, included a 7000-L storage tank for the primary dialysis acid and a 4000-L tank for lower calcium concentration acid. A pressurised piped delivery system delivers acid directly to the dialysis stations.

#### Outcomes

*Environmental Benefits:* This change eliminated 29,540 6L canisters, which equates to 4.2 tonnes of high-density polyethylene plastic waste annually. Reduced acid and plastic wastage was estimated to save 16.03 tonnes of carbon dioxide equivalent in 2011 [17]. Using calculations published by Murcutt et al. in 2024, which utilise updated carbon emission factors and account for transport emissions, the 40-station haemodialysis unit would save 45.12 tonnes of carbon dioxide equivalent per year.

*Cost Savings:* Annual savings calculated in 2012 were £23,072, with £19,372 saved from reduced acid wastage and £3,700 from decreased waste disposal costs [17]. The current price of £0.50/litre for 1:44 acid concentrate would generate savings of £25,071 annually. Additionally, reductions in domestic disposal would generate a saving of £438.56 annually.

*Operational Improvements:* Every one of the 29,540 (7.2 kg) canisters requires lifting twice to get from storage onto a trolley and subsequently to the dialysis machine. Centralised dialysis acid delivery has removed 425,376 kg of staff lifting per year. There is also increased resilience to supply chain disruption with a three-week supply of acid concentrate available at any given time [17].

### 1:44 acid concentrate

In 2011, alongside the switch to centralised dialysis acid delivery, the unit also changed from using 1:34 to 1:44 dilution acid concentrate. Both concentrates ultimately produce the same dialysate, as the haemodialysis machine dilutes each according to the pre-programmed ratio.

#### Outcomes

*Environmental Benefits:* The switch to 1:44 acid concentrate reduces the volume of acid concentrate required per treatment, thereby lowering transportation and storage requirements [18, 19]. This would reduce greenhouse gas emissions by approximately 1.48 tonnes carbon dioxide equivalent annually. Furthermore, the 4.7L 1:44 plastic canister requires 36 g less plastic to manufacture and dispose of than the 6L 1:34 canister, resulting in a further 0.57 tonne of carbon dioxide equivalent savings.

*Cost Savings:* There were no significant financial savings associated with the switch. However, the reduction in plastic waste associated with canister usage potentially reduced disposal costs.

*Operational Improvements:* The adoption of 4.7L canisters reduced the storage space required. As these canisters also weighed less, this further reduced the volume and weight of acid supplies that the staff needed to move.

### Dialysate autoflow function

The AutoFlow® function, available on Fresenius haemodialysis machines, automatically adapts the dialysate flow (Qd) to the effective blood flow (Qb). In 2013, the unit adjusted Qd to an Autoflow® ratio of 1.5 times Qb. Prior to this, the dialysate flow was either 500 ml/min or 800 ml/min. With the new setting, a Qb of 400 ml/min would now have a Qd of 600 ml/min instead of the previous 800 ml/min [20].

#### Outcomes

*Environmental Benefits:* The unit achieved a 9% reduction in water and acid concentrate usage in 2013, with estimated annual water savings of 1.14 million litres and greenhouse gas savings of 3.71 tonnes carbon dioxide equivalent [20]. We estimate a saving of 10.49 tonnes carbon dioxide equivalent annually using updated 2024 greenhouse gas factors.

*Cost Savings*: There was an annual saving of £11,524 due to less water, acid concentrate and bicarbonate consumption [20]. Using updated 2024 pricing, the unit would benefit from savings of £17,659 annually.

### Energy-efficient lighting

In 2012, the unit utilised its £5,000 British Journal of Renal Medicine (BJRM) Innovation in Renal Medicine Award 2011 monies to replace 85 T8 fluorescent lights with more energy-efficient T5 fittings in the renal unit [21]. The average energy saving per individual light bulb change was 36 kWh per year, with estimated annual savings of 2.3 tonnes carbon dioxide equivalent and £612 financially. The unit also experienced improved lighting levels, which enhanced the working environment, and there were reduced maintenance needs due to the longer life of T5 fittings [21].

### Incremental and decremental haemodialysis

An incremental and decremental haemodialysis program was implemented in 2020. The incremental program tailors dialysis frequency and duration to each patient's individual needs, starting with twice weekly instead of the conventional thrice weekly four-hour haemodialysis sessions and gradually increasing either haemodialysis time or the number of haemodialysis sessions as necessary. Similarly, for patients with recovering kidney function or those who may be approaching end-of-life care and can only tolerate less intensive regimens, the prescribed haemodialysis dose can be decreased. The unit has had an average of 25 patients on incremental or decremental haemodialysis at any given time since 2020.

#### Outcomes

*Environmental Benefits:* With a reduced frequency and/or duration of haemodialysis, incremental and decremental dialysis reduces the annual resource usage and amount of waste produced per patient. Twenty-five patients on twice-weekly dialysis would produce 28.2 tonnes less carbon dioxide equivalent annually.

*Cost Savings:* A multicentre feasibility randomised controlled trial assessing the impact of incremental versus conventional initiation of haemodialysis found that the median health care provider costs were £19,875 in the incremental arm compared to £26,125 in the standard thrice weekly arm due to reduced transport, haemodialysis, and adverse events costs [22]. Based on these data, we estimate a saving of £156,250 annually.

*Patient Care:* A personalised haemodialysis treatment schedule can enhance patients' quality of life by minimising time spent on dialysis. The unused mid-week dialysis slot allows the unit to flex its dialysis capacity when needed to accommodate additional haemodialysis or ultrafiltration needs when required.

**Fig. 1:**
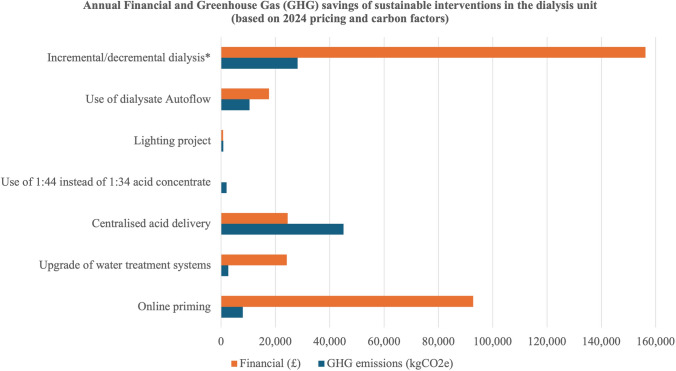
Annual Financial and Greenhouse Gas (GHG) savings of sustainable interventions in the dialysis unit (based on 2024 pricing and carbon factors). *Based on an average of 25 patients per year on twice weekly haemodialysis

Table 1 provides a list of all implemented changes alongside greenhouse gas and financial savings. The estimates in haemodialysis-related interventions are based on a 40-bed haemodialysis unit. Figure 1 illustrates the financial and greenhouse gas savings for 2024.

**Table 1:** Summary of sustainable interventions in our renal unit from 2007–2024

Interventions	Capital outlay costs	Year introduced	Financial savings per year (estimated / actual) and estimated greenhouse gas (CO_2_e) savings per year*	Cumulative estimated savings to date
Electronic consultations via: 1) E-consultation and E-referrals via SystmOne® as an alternative to paper hospital referral for advice and referrals 2) NHSAttend Anywhere 3) Virtual Renal Metabolic Stone 4) Young Adult Transition Clinics	None^†^	2007 and 2020	Financial: Unable to quantify financial savings accurately CO_2_e: 22 kg saved per outpatient visit avoided	CO_2_e: 165.00 tonnes (approximately 7500 appointments over 18 years)
Renal Unit Hub via Systm One ® - Two-way sharing of Electronic Health Records between the renal unit and general practice	None^†^	2010	Unable to accurately quantify financial and CO_2_e savings. However, this initiative resulted in reduced face-to-face hospital attendances for blood test monitoring and reduced paper communications to and from primary care teams (thousands of communications per annum)	
Use of online priming on haemodialysis machines (avoiding saline bags)	None	2010	Financial: £92,851 (Estimated) CO_2_e: 8.04 tonnes	Financial: £1,392,765 (15 years) CO_2_e: 120.6 tonnes (15 years)
Upgrade of water treatment systems in the haemodialysis unit	£60,000	2011	Financial: £18,000 (Actual, 2012) Financial: £24,240 (Estimated, 2024) CO_2_e: 8.42 tonnes (2012) CO_2_e: 2.71 tonnes (2024)	Financial: £252,000–339,360 (14 years) CO_2_e: 37.94–117.88 tonnes (14 years)
Central delivery of acid for haemodialysis	£43,900	2011	Financial: £23,072 (Actual, 2012) Financial: £25,509 (Estimated, 2024) CO_2_e: 45.12 tonnes	Financial: £323,008–357,126 (14 years) CO_2_e: 586.56 (13 years)
Use of 1:44 haemodialysis acid concentrate solution	None	2011	Financial: £0 (Actual) CO_2_e: 2.05 tonnes	Financial: £0 (14 years) CO_2_e: 28.7 tonnes (14 years)
Lighting Project—installation of 85 fluorescent 'T5' light fittings to replace the order ‘T8’ light fittings	£5,000	2012	Financial: £612 (Estimated, 2012) Financial: £765 (Estimated, 2024) CO_2_e: 2.30 tonnes (2012) CO_2_e: 0.85 tonnes (2024)	Financial: £7,956–9,945 (13 years) CO_2_e: 11.05- 29.9 tonnes (13 years)
Use of dialysate Autoflow facility	None	2013	Financial: £11,524 (Estimated) Financial: £17,659 (Estimated, 2024) CO_2_e: 3.71 tonnes (2013) CO_2_e: 10.49 tonnes (2024)	Financial: £138,288 – 211,903 (12 years) CO_2_e: 44.52–125.88 tonnes (12 years)
Incremental and decremental haemodialysis (Average 25 patients per year on twice weekly haemodialysis)	None	2020	Financial: £156,250 CO_2_e: 28.21 tonnes	Financial: £781,250 (5 years) CO_2_e: 141.05 tonnes (5 years)

CO_2_e: Carbon dioxide equivalent, GHG: Greenhouse gas, NHS: National Health Service

^*^GHG (CO_2_e) estimates provided in this table are complemented by calculations supplied in the supplementary material

^†^There were no capital costs to the department as the department worked in collaboration with the service developer to produce the prototype system

## Discussion

The renal unit’s initiatives illustrate a proactive approach to reducing the environmental footprint of kidney care while ensuring financial efficiency and maintaining high standards of patient care. By implementing various interventions, including technological upgrades, process optimisations, and resource-saving strategies, the unit has effectively addressed several sustainability challenges in healthcare.

The financial analysis demonstrates that many of these interventions not only contribute to environmental sustainability but also provide cost savings in the long term. Online priming, water treatment system upgrades, centralisation of dialysate acid concentrate delivery, and incremental dialysis programme each generated considerable annual savings in water, acid concentrate, consumables and material costs. These results underscore the importance of viewing environmental sustainability as an investment, where subsequent savings and increased resilience in healthcare operations can justify high capital costs. In addition, interventions such as using centralised dialysis acid delivery systems improve operational efficiency. As described by Murcutt et al. (2024), centralised dialysis acid delivery systems reduce the amount of waste generated per dialysis session and lessen staff workload by minimising manual handling requirements, and saving time during machine setup and cleanup [23]. A dialysis unit in Italy that implemented centralised dialysis acid delivery in 2022 reported similar results, with nurses noting an increase in time dedicated to their duties due to the lighter workload from reduced manual handling requirements [24].

The economic burden of providing dialysis care in the United Kingdom was calculated to be £1.05 billion in 2023 or 0.53% of the NHS budget [25]. Improvements in resource usage, such as described in this paper in renal services, could reduce the financial strain on the NHS or at least allow the expansion of services to meet increasing demand.

Each intervention was monitored to ensure that environmental benefits were achieved without compromising on patient care. Using the dialysate autoflow facility in haemodialysis treatments resulted in reduced acid and water consumption without negatively impacting dialysis adequacy. Alayoud et al. (2012) demonstrated that an autoflow ratio between 1.2 and 1.5 effectively achieves the desired dialysis dose, suggesting that increasing Qd to offset low Qb may have limited value [26]. Albalate et al. (2015) observed that increasing Qd from 400 to 500 mL/min improved the Kt value by only 4%, and from 500 to 700 mL/min by 3%. They noted that a comparable increase in dialysis adequacy could be achieved by extending treatment time by a few minutes, which still reduces overall dialysate and water consumption [27].

Similarly, incremental and decremental dialysis approaches provide more tailored treatment regimens for patients, reducing unnecessary dialysate use and supporting a patient-centred approach to care. Such practices align with the growing focus on personalised medicine, where resource usage is adapted to patient needs, reducing both environmental and physical burdens on patients. A meta-analysis demonstrated that incremental initiation of haemodialysis does not confer a greater mortality risk compared with standard treatment, and hospitalisation episodes may be reduced [28].

The use of e-consultations and virtual appointments also offers substantial benefits to patients. A study published by The Strategy Unit found that virtual appointments provide the opportunity to mitigate the negative socio-economic impacts associated with traditional outpatient services. This is due to reduced work absenteeism (time off to attend and travel to outpatient appointments) and the direct costs incurred by individuals from travel and parking [29]. There was also a reduction in waiting times for specialist nephrology advice. In Bradford, the wait for specialist advice decreased from 55 to 7 days. A 2020 study in East London found similarly that the wait for specialist advice decreased from 64 to 6 days, boosting GP confidence in managing CKD and enhancing patient satisfaction [30].

The adoption of sustainable practices in kidney care represents a step toward aligning clinical care with the pressing need to address climate change and resource efficiency. If implemented at scale, such initiatives could lead to substantial reductions in carbon emissions and resource utilisation while potentially generating long-term financial savings. It is difficult to accurately extrapolate the financial and greenhouse gas savings of these practices at a national or global scale without robust baseline data on current services. Based on our assumptions, implementing similar sustainable initiatives across kidney units in the UK could potentially achieve savings of almost 5,000 tonnes of carbon dioxide equivalent emissions per year (Supplementary material). Challenges of implementing sustainable initiatives, such as variation in infrastructure, competing operational priorities, lack of incentives, limited staff capacity, lack of knowledge and resistance to change may hinder implementation [31]. Overcoming these barriers requires investment, leadership commitments and integration of sustainability into everyday clinical and procurement decision-making. The KitNewCare consortium aims to develop a benchmarking tool that enables the mapping of sustainability in kidney care, thereby deriving solutions targeted at greenhouse gas hotspots within the kidney care sector. This approach will provide evidence-based scalable solutions that can be employed more widely [32].

It is also important to consider how sustainable innovations may affect healthcare equity. Green technologies may require significant capital investment and may not be accessible to all centres. Without deliberate planning, sustainability efforts risk reinforcing existing inequalities. Embedding sustainable practices within healthcare systems, such as establishing a sustainable procurement pathway under development by the Global Environmental Evolution in Nephrology and Kidney Care (GREEN-K), offers a more equitable and replicable approach [33]. This strategy allows for consistent implementation across diverse regions and supports standardisation of care while advancing environmental goals. Additionally, aligning local efforts with broader global health policy frameworks, such as the World Health Organisation’s Quality Criteria for Health National Adaptation Plans and the United Nations Sustainable Development Goals, can strengthen the case for institutional and government support. In the UK, the Greener NHS programme provides a strategic direction aimed at achieving net zero emissions. However, despite these high-level commitments, many policies lack enforcement mechanisms or dedicated funding streams, which can limit the pace of change, especially for under-resourced areas.

The hybrid carbon footprinting approach was utilised because several interventions were implemented years earlier without contemporaneous tracking of carbon or financial savings. A purely top-down approach, which relies solely on aggregated data, would not capture the specificity required for certain calculations. While a purely bottom-up approach would yield a more accurate result, it necessitates detailed measurements of every component, which was impractical given the retrospective nature of the analysis. By blending these methodologies, we ensured that the carbon footprint estimates were viable within the constraints of available data.

In addition, some estimates may vary due to differences in operational practices and infrastructure between Bradford and the comparator institutions and the difference in carbon emissions factors, which are updated yearly by DESNZ/DEFRA. These limitations are noted in the analysis.

We acknowledge that the interventions described are all based in a single centre in the UK and may not be replicable in other settings, depending on financial resources and current infrastructure. As this study aims to provide kidney care service providers with successful sustainable initiatives in a renal unit and to allow readers to identify the environmental and financial savings associated with sustainability, this method provided a reasonable best estimate.

This study did not assess any specific consequences of switching to e-consultations compared to in-person review, e.g. rates of hospitalisation. However, interviews conducted with GPs and patients were supportive of this change to improve service efficiency and reduce unnecessary hospital appointments [11].

## Conclusion

The estimated cumulative savings surpass 1000 tonnes of carbon dioxide equivalent and £2.8 million over a 17-year period in our centre. While interventions for environmental sustainability may require upfront costs and staff time and effort, the dividends are long-term benefits such as environmental protection, financial savings, improved quality of care, greater staff satisfaction, and increased service resilience. The Bradford Renal Sustainability Group continues to explore innovative methods to lower the carbon footprint of kidney care. This includes collaboration with key organisations such as the Green Nephrology Group in Newcastle upon Tyne Hospitals NHS Foundation Trust, Sustainable Healthcare Coalition, Kidney Quality Improvement Partnership, NHS Blood and Transplant, Centre for Sustainable Healthcare and Health Foundation in the use of the online in-centre haemodialysis carbon calculator, reducing pharmaceutical and consumable waste and in streamlining the living kidney donor assessment pathway.

There is a critical need to amplify and replicate these initiatives on a global scale. International collaboratives such as the GREEN-K provide vital platforms for shared learning and standard setting. In particular, the adoption of digital technologies and innovative solutions will be pivotal in scaling sustainable kidney care across diverse healthcare systems. By sharing our experiences and outcomes, we hope this may assist other institutions in integrating green initiatives into everyday service planning.

## Supplementary Information

Below is the link to the electronic supplementary material.

Supplementary file1 (DOCX 53 KB)

## Data Availability

All data generated or analysed during this study are included in this published article and its supplementary information files.

## Glossary

CAD (Centralised acid delivery): Centralised acid delivery is a system used in dialysis units to supply acid concentrate through a centralised distribution system rather than individual canisters.
CO_2_e (Carbon dioxide equivalent): Carbon dioxide equivalent is a standard unit used to express global warming potential of different greenhouse gases in terms of the amount of CO_2_ that would have the same impact. For example, one kg of methane has a much greater warming potential than one kg of CO_2_, so it is expressed in CO_2_e to allow comparisons across different gases. This metric is commonly used in carbon footprint assessments.
EHR (Electronic Health Record): An EHR is a digital version of a patient’s paper chart. It contains medical diagnoses, medications, clinical encounters and test results.
GHG (Greenhouse gas): Greenhouse gases are gases in the atmosphere that traps heat and contribute to the greenhouse effect. Common GHGs include carbon dioxide, methane and nitrous oxide.
