# Supplementary material for: Sustainable health care in a renal centre - carbon saving is coupled with cost-efficiency
Source: J Nephrol. 2025 Jul 23;38(8):2321–31. doi: 10.1007/s40620-025-02354-x (PMC12630188; doi:10.1007/s40620-025-02354-x)
Supplement: Supplementary file 1 — Supplementary file1 (DOCX 53 KB) [file 40620_2025_2354_MOESM1_ESM.docx]

**Title:** Sustainable health care in a renal centre - carbon saving is coupled with cost-efficiency

**Authors:** ^1^Stephanie Mei Yann Choo, ^3^Gareth Murcutt, ^3^Ingeborg Steinbach,  ^4^John Stoves

^1^ Department of Renal Medicine, St James’s University Hospital, Beckett St, Harehills, Leeds LS9 7TF

^2^ UCL Department of Renal Medicine, Royal Free Hospital London, London NW3 2QG, UK

^3^ Centre for Sustainable Healthcare, 8 King Edward Street, Oxford OX1 4HL, UK

^4^ Department of Renal Medicine, Bradford Teaching Hospitals NHS Trust, Duckworth Ln, Bradford BD9 6RJ

**Supplementary Material:**

Words 1920

Tables 10

References 9

**Introduction**

This section describes the greenhouse gas (GHG) savings of each individual intervention described in the main paper.

**e-consultations calculations**

One outpatient face-to-face appointment is estimated to emit 22kgCO_2_e per visit [1, 2]. Hence, the GHG savings were calculated as 7,500 avoided appointments multiplied by the emission factors (7500 x 22 kgCO_2_e = 165,000kgCO_2_e).

**Online priming calculations**

Online priming negates the use of individual 0.9% sodium chloride (NaCl) infusion bags and connecting tubes. The calculations of the removal of these items are as detailed below.

Sodium Chloride Component

Carbon Footprint for NaCl: 0.3 kg CO₂e per kilogram (Source: Murcutt. et al 2024 [3])

Amount of sodium chloride in 500 mL Solution: 0.009kg

Emissions: 0.009 x 0.3kg CO₂e/kg = 0.0027 kgCO₂e (2.70gCO_2_e)

Water Component

Water supply emission factor: 0.15311 kgCO₂e per m^3^ (Source: DESNZ/DEFRA 2024 [4])

Water treatment emission factor: 0.18574 kgCO₂e per m^3^ (Source: DESNZ/DEFRA 2024 [4])

Total water emission factor: 0.15311 + 0.18574 = 0.33885 kgCO₂e per m^3^

Volume of water in 1000 mL 0.9% sodium chloride solution: 1L = 0.001m^3^

Water emissions: 0.001m^3^ x 0.33885kgCO₂e/m^3^ = 0.00034 kgCO₂e (0.34gCO2e)

Packaging component of 0.9% NaCl 500ml infusion bag

The 0.9% NaCl infusion is supplied in a plastic bag covered with an outer plastic packaging which is made from specially formulated polyvinylchloride (PVC) [5]. DESNZ/DEFRA 2024 emission factor for PVC and waste stream emission factors from Rizan et al. (2021) were used [4, 6].

Supplementary table 1: Carbon emissions of packaging material of a single 0.9% sodium chloride 1000ml infusion bag

|  | Material | Weight (kg) | Production emission factor | Waste stream and emission factor | Total emissions |
| --- | --- | --- | --- | --- | --- |
| Inner packaging | PVC | 0.020 | 2.936 kgCO_2_e/kg | Non-infectious offensive waste: 0.249 kgCO_2_e/kg | 0.064 |
| Outer packaging | PVC | 0.010 | 2.936 kgCO_2_e/kg | Domestic waste : 0.172 kgCO_2_e/ kg | 0.031 |
| Total carbon emissions of packaging material of 0.9% NaCl 500ml infusion bag | | | | | 0.095kgCO_2_e (95.00gCO_2_e) |

Intravenous infusion giving set component

The emission factor of an intravenous infusion giving set was calculated as 116.82gCO_2_e in Rizan et al’s study published in 2023 [7].

Supplementary table 2: Total carbon and financial reductions by using online priming of haemodialysis machines with dialysate

| **Components** | | **Carbon emissions** |
| --- | --- | --- |
| 0.9% sodium chloride | NaCl | 2.70 gCO_2_e |
|  | Water | 0.34 gCO_2_e |
|  | Packaging | 95.00 gCO_2_e |
| Intravenous infusion giving set | | 116.82 gCO_2_e |
| Total carbon reduction per session | | 214.86 gCO_2_e/session |
| Total carbon emissions reduction in a year (37440 haemodialysis session) | | 8044.36 kgCO2e |

**Upgrade of water treatment system calculations.**

In 2012, the centre calculated the water savings to be 8 million litres of water annually after the upgrade. The conversion factors for water supply and treatment published by DEFRA in 2012 were significantly higher compared to 2024; hence, an initial saving of 8.42 tonnes CO_2_e was calculated. As DESNZ/DEFRA refines its methodologies and updates its conversion factors yearly, we have used the updated DESNZ/DEFRA 2024 conversion factors for water, as detailed below, to provide an updated estimate.

Water supply and treatment emission factor: 0.15311 + 0.18574 = 0.33885 kgCO₂e per m^3^

Total carbon reduction: 8000 m^3^ x 0.33885 = 2711 kgCO_2_e

**Central acid delivery calculations**

Murcutt et al.(2024) published the greenhouse gas savings for a 30-bed dialysis unit utilising central acid delivery (CAD) as opposed to using individual 5L containers in 2024. A 30-station unit operating a maximum capacity of 3 shifts per day, 6 days per week could realise an approximate total reduction of 33,841 kgCO_2_e/year [3]. By scaling this to a 40-station unit operating at a similar maximum capacity, this would amount to 45,121 kgCO_2_e/year.

**Change from 1:34 to 1:44 acid concentrate calculations**

The average blood flow of patients dialysing in our unit is 350ml/min. As we use an Autoflow function of 1.2 (40% of patients on haemodiafiltration) or 1.5 (60% of patients on haemodialysis), this would give us an average dialysate flow rate of 483 ml/min. This allowed us to calculate the average consumption of acid concentrate to be 3.312L in 1:34 acid and 2.596L in 1:44 acid.

The total carbon emissions of changing from 1:34 to 1:44 acid concentrate is calculated by adding the difference in production and transport emissions and plastic canister waste. The production emission factor for 1:44 acid concentrate of 1548kgCO_2_e per 10,000L batch of acid concentrate was derived from Murcutt et al’s 2024 publication and amended using DENSZ/DEFRA 2024 GHG factors [3]. We calculated the carbon footprint of manufacturing 1:34 dialysate acid concentrate using similar methods with data provided by the Original Equipment Manufacturer, Fresenius Medical Care, UK Ltd.

Supplementary table 3: Estimated carbon footprint of the manufacture of 10,000 litres of 1:44 dialysate acid concentrate

| **Item** | **Quantity** | **Carbon emission factor (kgCO_2_e/unit)** | **Total Emissions (kgCO_2_e)** |
| --- | --- | --- | --- |
| Water | 13.254 m^3^ | 0.15311 kgCO_2_e/m^3^ | 2.03 |
| NaCl | 2924 kg | 0.3 kgCO_2_e/kg | 877.20 |
| Glucose | 512 kg | 1.1 kgCO_2_e/kg | 563.20 |
| Acetic Acid | 103 kg | 0.61 kgCO_2_e/kg | 62.83 |
| Energy | 160 kWh* | 0.27522 kgCO_2_e/kWh | 44.04 |
| Waste water | 4.418 m^3^ | 0.18574 kgCO_2_e/m^3^ | 0.82 |
| Total concentrate production emissions (10000L batch) | | | 1550.12 |

*Based on 0.016kWh electricity consumed per 1L of acid concentrate manufactured.

Supplementary table 4: Estimated carbon footprint of the manufacture of 12,900 litres of 1:34 dialysate acid concentrate

| **Item** | **Quantity** | **Carbon emission factor (kgCO_2_e/unit)** | **Total Emissions (kgCO_2_e)** |
| --- | --- | --- | --- |
| Water | 16.548 m^3^ | 0.15311 kgCO_2_e/m^3^ | 2.53 |
| NaCl | 2924 kg | 0.3 kgCO_2_e/kg | 877.20 |
| Glucose | 512 kg | 1.1 kgCO_2_e/kg | 563.20 |
| Acetic Acid | 103 kg | 0.61 kgCO_2_e/kg | 62.83 |
| Energy | 206.4 kWh* | 0.27522 kgCO_2_e/m^3^ | 56.81 |
| Waste water | 5.516 m^3^ | 0.18574 | 1.02 |
| Total concentrate production emissions (12900L batch) | | | 1563.59 |

*Based on 0.016kWh electricity consumed per 1L of acid concentrate manufactured.

Supplementary table 5: Volume, weight and production carbon emissions difference using 1:34 vs 1:44 acid concentrate in dialysis

| **Description** | **1:34** | **1:44** | **Difference** |
| --- | --- | --- | --- |
| Volume of acid concentrate required per session | 3.312L | 2.576L | 0.736 |
| Volume of acid concentrate required per year | 124001L | 96445L | 27556L |
| Weight of acid concentrate per L | 1.168kg | 1.220kg | -1.425kg |
| Weight of acid concentrate required per year | 144833kg | 117663kg | 27170kg |
| Production emissions | 15034kgCO_2_e | 14949kgCO_2_e | 85kgCO_2_e |

Supplementary table 6: Savings in transport greenhouse gas emissions as a result of switching from 1:34 to 1:44 acid concentrate

| **HGV Diesel (7.5 - 17 tonne) Average Laden** | **Emissions factor (kgCO_2_e per tonne.km)** | **Tonne** | **km** | **tonne.km** | **Total transport emission savings (kgCO_2_e)** |
| --- | --- | --- | --- | --- | --- |
| Transport (Freight) | 0.38023 | 27.17 | 109 | 2961.53 | 1126.06 |
| Transport (Well-to-tank) | 0.09246 | 27.17 | 109 | 2961.53 | 273.82 |
| Total | | | | | 1399.89 |

Plastic canister savings

The 4.7L 1:44 empty plastic canister weighs 260g, while a 6L 1:34 empty plastic canister weighs 296g. This results in a reduction of 36g of high-density polyethylene plastic waste per canister. An average of 94 canisters are used per week for patients not on central acid delivery.

Number of canisters used annually: 4888

Weight difference between 1:44 (4.7L) and 1:34 (6L) empty plastic canisters: 0.036kg

Total weight difference in plastic canister usage annually: 175.97kg

HDPE production emission factor: 3.086 kgCO_2_e/kg

Domestic waste emission factor: 0.172 kgCO_2_e/ kg

Total plastic emission savings: 175.97kg x (3.086+0.172)kgCO_2_e/kg = 573.31kgCO_2_e

**Use of dialysate autoflow facility calculations**

The initial savings of this intervention in 2013 are published on the Centre for Sustainable Healthcare website . Unfortunately, the detailed data for the calculations completed in 2013 were not fully available. However, to estimate the savings using 2024 DESNZ/DEFRA 2024 factors, we have estimated the environmental saving of switching from an average dialysate flow rate (Qd) of 620ml/min to an Autoflow rate of 1.5 times the average blood flow rate of 350ml/min, which gives a new Qd of 525ml/min, saving 95ml/min or 22.8L of dialysate in a 4 hours dialysis session. The average Qd of 620ml/min is based on the assumption from local data that the proportion of patients dialysing with a Qd of 500ml/min and 800ml/min were 60% and 40%, respectively.

Supplementary table 7: Estimated greenhouse gas and financial savings switching from 500ml/min to Autoflow rate of 1.5.

| **Saving** | **Per min** | **Per dialysis session** | **Per annum** | **Emission factor** | **GHG reduction per annum (kgCO_2_e)** | **Financial saving per annum (£)** |
| --- | --- | --- | --- | --- | --- | --- |
| Water | 0.133L* | 31.92L | 1195085L (1195m^3^) | 0.33885 kgCO₂e per m^3^ | 404.95 | 3621 |
| Electricity | - | 0.64kWh | 23901.70kWh | 0.27522 kgCO₂e per kWh | 6572.97 | 5975 |
| Acid concentrate production | 0.002L^†^ | 0.507L | 16124L | 0.155 kgCO₂e  per L | 2499.24 | 8062 |
| Acid concentrate transport | - | - | 2144.19 tonne.km | 0.47269 kgCO₂e per tonne.km | 1013.54 | - |
| Total savings | | | | | 10490.7 | 17659 |

GHG = Greenhouse gas

*The water consumption is estimated at 133ml/min to account for the additional 40% reject water produced during the production of 95ml/min ultrapure water.

^†^The acid concentrate used for this calculation is 1:44 dilution acid concentrate, with 85% of patients being on central acid delivery and the rest of the patients dialysing with dialysate concentrate in canisters. The savings for acid concentrate using canisters were not calculated as any excess acid remaining in the canister will be disposed of.

**Lighting project calculations**

A total of 85 light fittings were changed in the renal unit offices and wards. The average energy saving per individual light was estimated at 36 kilowatt hours (kWh) per year. The calculations for this intervention in 2012 are published on the Centre for Sustainable Healthcare website. The calculations using DESNZ/DEFRA 2024 factors are as detailed below.

Supplementary table 8: United Kingdom (UK) electricity carbon emissions factor

| **Activity** | **Emission factor (kgCO2e per kWh)** |
| --- | --- |
| UK electricity generation | 0.20705 |
| UK electricity transmission and distribution loss | 0.0183 |
| Well-to-tank UK electricity (generation) | 0.0459 |
| Well-to-tank UK electricity (transmission and distribution loss) | 0.00397 |
| Total UK electricity emission factor | 0.27522 |

Estimated annual GHG saving of lighting change: 85 (light fittings) x 36 kWh x 0.27522 = 852 kgCO_2_e.

**Incremental haemodialysis calculations**

Connor et al. published that in-centre haemodialysis has a carbon footprint of 3.8 tonne CO_2_e per patient per year, giving an estimate of 24.3 kgCO_2_e per haemodialysis session. A more recent study by Newcastle calculated that a single haemodialysis session contributes to 21.7 kgCO_2_e [9]. Using the more conservative estimate, 25 patients on twice-weekly dialysis would have 1300 fewer haemodialysis sessions per year. Thus generating 28.21 tonnes less CO_2_e annually.

**Financial savings**

Unless stated otherwise, the cost of utilities or consumables used to calculate financial savings in this paper are summarised in Supplementary Table 9.

Supplementary table 9: Cost of utilities and consumables

| **Item** | **Costs (**£) |
| --- | --- |
| Water supply treatment and sewerage (£ per m^3^) | 3.03 |
| Electricity (£ per kWh) | 0.25 |
| Acid concentrate (£ per L) | 0.5 |
| Domestic waste disposal (£ per tonne) | 109.42 |
| 0.9% sodium chloride infusion bag (1 litre) | 0.96 |
| Intravenous infusion giving set (1 set) | 1.52 |

**Estimated annual GHG savings if sustainable interventions described in our centre are implemented across the UK**

For this calculation, we assume the average size of a main haemodialysis unit is 30 stations and a satellite unit is 10 stations operating three sessions a day for six days a week. Our calculations are also based on 72 main dialysis units and 204 satellite units across the UK. Our assumptions on the uptake of infrastructure and process innovations are based on the modelling described by Limb (2013) [9].

Supplementary table 10: Estimated annual GHG savings in the UK

|  | **Savings in in a single centre (Bradford)** | | **Savings in a single main or satellite unit** | |
| --- | --- | --- | --- | --- |
|  | Number per year | GHG per year (tCO2e) | GHG per year (tCO2e) | |
|  | 40 stations | | Main  (30 stations) | Satellite  (10 stations) |
| Central Acid Delivery |  | 45.12 | 33.84 | 11.28 |
| Lighting project |  | 0.85 | 0.64 | 0.21 |
| *Total infrastructure changes* |  | *45.97* | *34.48* | *11.49* |
|  | | | | |
| Virtual consultations | 1,200 | 26.4 | 19.80 | 6.60 |
| Online priming |  | 8.04 | 6.03 | 2.01 |
| Switching to 1:44 |  | 2.05 | 1.54 | 0.51 |
| Autoflow |  | 10.49 | 7.87 | 2.62 |
| Incremental/decremental HD |  | 28.21 | 21.16 | 7.05 |
| *Total process changes* |  | *75.19* | *56.39* | *18.80* |
| Total |  | 121.16 | 90.87 | 30.29 |
| Assumption: 20% main dialysis units already implemented innovations, out of the remaining 80%, 30% implement the infrastructure innovations and 60% process innovations | | | 2,544.70 |  |
| Assumption: 20% of satellite units have already implemented innovations, out of 80%, 30% implement the infrastructure innovations and 60% process innovations | | |  | 2,403.32 |
| **Total** | | | | **4,948.02** |

**References**

1. Connor A, Lillywhite R, Cooke MW (2010) The carbon footprint of a renal service in the United Kingdom. QJM 103:965–975. https://doi.org/10.1093/qjmed/hcq150

2. Tongue B, Moore R (2022) Business Case Carbon Impact Tooling database V3.01, tab ‘healthcare events’.

3. Murcutt G, Hillson R, Goodlad C, Davenport A (2024) Reducing the carbon footprint for a 30-bed haemodialysis unit by changing the delivery of acid concentrate supplied by individual 5 L containers to a central delivery system. J Nephrol 37:1949–1955. https://doi.org/10.1007/s40620-024-02073-9

4. Department for Energy Security and Net Zero (DESNZ), The Department for Environment F and RA (DEFRA) (2024) Greenhouse gas reporting: conversion factors 2024

5. (2018) Sodium chloride injection, USP in VIAFLEX plastic container. Baxter Healthcare Corporation

6. Rizan C, Bhutta MF, Reed M, Lillywhite R (2021) The carbon footprint of waste streams in a UK hospital. J Clean Prod 286:125446. https://doi.org/10.1016/j.jclepro.2020.125446

7. Rizan C, Lillywhite R, Reed M, Bhutta MF (2023) The carbon footprint of products used in five common surgical operations: identifying contributing products and processes. J R Soc Med 116:199–213. https://doi.org/10.1177/01410768231166135

8. Costelloe T, Collins M, Shah J (2022) In-Centre Haemodialysis at Newcastle upon Tyne Hospital

9. Limb, M (2013). NHS could save £1bn by adopting green strategies used in kidney units. BMJ (Clinical Research Ed.), 346:f588. https://doi.org/10.1136/bmj.f588
